# Influence of Silanization Treatment of Sponge Gourd (*Luffa cylindrica*) Fibers on the Reinforcement of Polyester Composites: A Brief Report

**DOI:** 10.3390/polym14163311

**Published:** 2022-08-15

**Authors:** Eduarda Chiabai Rodrigues de Melo, Mayara de Oliveira Camillo, Paulo Roberto Correia Marcelino, Roseméri Barbosa dos Santos da Silva, Thierry Colares Firmino, Bárbara Ferreira de Oliveira, Demetrius Profeti, Artur Camposo Pereira, Sergio Neves Monteiro, Michel Picanço Oliveira

**Affiliations:** 1 Forest and Wood Sciences Department, Federal University of Espírito Santo, Jeronimo Monteiro 29550-000, Brazil; 2 Advanced Materials Department, Northern Fluminense State University, Campos dos Goytacazes 28013-602, Brazil; 3 Chemistry and Physics Department, Federal University of Espírito Santo, Alto Universitário, sn., Porto Alegre 29500-000, Brazil; 4 Materials Science Program, Military Institute of Engineering—IME, Praça General Tibúrcio 80, Urca, Rio de Janeiro 22290-270, Brazil

**Keywords:** polymer, lignocellulosic fiber, sponge gourd, chemical treatment, mechanical proprieties

## Abstract

Natural lignocellulosic fibers (NLFs) have been extensively investigated and applied as reinforcements for polymers composites owing to improved properties associated with their cost-effectiveness and their sustainable characteristics as compared to synthetic fibers. However, an intrinsic difficulty of the hydrophilic NFL adhesion to a hydrophobic polymer matrix is still a major limitation, which might be overcome via fiber surface treatments. Among the less-known NLFs, sponge gourd *(Lufta cylindrica*) is a promising reinforcement for polymer composites owing to its natural network of intertwined fibers. The present work investigated for the first time the influence of a chemical treatment using silane as a coupling agent for 30 wt.% sponge gourd incorporated into a polyester matrix composite. The novel composite performance was compared with that of an untreated fiber composite via X-ray diffraction (XRD), Fourier transformed infrared spectroscopy (FTIR), Charpy impact tests, and thermogravimetric analyses (TGA). The XRD results revealed that the silanization increased the crystallinity index by 37%, which attests to the effective fiber–matrix interaction stretching of the C-H bond, as observed in its FTIR band. The silanization also increased the mean impact resistance by 10%. Although the temperatures associated with the beginning of the thermal degradation by the TGA were not affected, both the silane-treated fibers and composite displayed less thermal degradation compared with the untreated fibers. The scanning electron microscopy and energy-dispersive X-ray spectroscopy (SEM/EDS) results disclosed an improved sponge gourd fiber morphology after the silanization, which caused greater adherence to the polyester matrix. These results revealed a promising novel composite compared with other NLF polymer composites in engineering applications.

## 1. Introduction

There has been an increase in the worldwide interest in the development of new sustainable materials for industrial applications, from packing to automotive parts. The motivation is focused on products that have less environmental impact and at the same time can meet the mechanical and structural requirements of the industries [1,2,3,4]. In this respect, fiber-reinforced polymer matrix composites are preferred materials for their comparatively low weight, high strength, and superior stiffness [5,6,7]. However, if the reinforcement is made of synthetic fibers, then the sustainable characteristic is compromised. In contrast to synthetic fibers, biodegradable and recyclable natural lignocellulosic fibers (NLFs) are currently being extensively used in polymer composites owing not only to their sustainability but also their other advantages, such as their lower cost and density and higher specific mechanical properties [8,9,10,11]. Indeed, numerous NLFs have in the past decades been investigated and industrially applied as reinforcements for composites with different polymer matrices [12,13,14,15].

**Figure 1:**
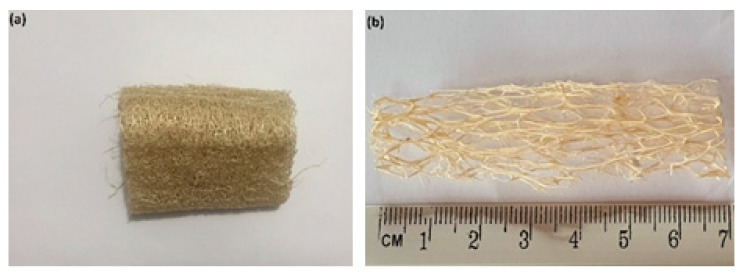
(**a**) Sponge gourd (*Luffa cylindrica*) fibers as received and (**b**) after cleaning and being used for reinforcement in composites.

Among the less known NFLs applied for composite reinforcement is the sponge gourd, also called a luffa, obtained from the dried fruit of *Luffa aegyptiaca*, a plant belonging to the cucurbitaceae species, cultivated in subtropical regions of Asia, Africa, and South America. The sponge gourd is naturally composed of interconnected fibers forming a cylindrical structure measuring ~50 mm in diameter and ~200 mm in length, as shown in Figure 1. Its chemical composition varies in the range of 57–74% cellulose, 14–30% hemicellulose, 10–20% lignin, and up to 12% other constituents such as ash and extractives [16,17]. Both due to its interconnected structure (Figure 1) and chemical composition, sponge gourd has been investigated as a possible reinforcement for polymer composites.

Table 1 presents the results reported for sponge gourd composites, which reveal the promising mechanical properties and favorable physical behavior [16].

The results in Table 1 shows the proprieties of composites with different matrices reinforced with sponge gourd. However, as with any NFL, the hydrophilic sponge gourd fiber displays reduced adhesion to a normally hydrophobic polymer matrix. A prior physical or chemical surface treatment might be performed to improve the fiber–polymer matrix adhesion [18,19]. Mercerization with NaOH is one of the most used alkaline pretreatments to expose reactive sites on the fiber surfaces [20,21]. Although improving the adhesion, mercerization also causes deeper damage to soft natural fibers, which impairs the overall composite behavior [22,23,24].

Silanization is an alternative to sponge gourd fibers that can be used to avoid the excessive damage caused by the strong alkaline reaction involved in mercerization. The chemical modification of the sponge gourd, provided by the silane coupling agent, results in a cross-linked network of surface fiber–polymer matrix covalent bonds [25]. This contributes to increasing the fiber–matrix compatibility by introducing functional groups that facilitate the mutual interaction.

In spite of the investigation conducted in other silanized NFLs reinforcing polymer composites [26,27,28,29], to our knowledge no work has yet investigated the effect of sponge gourd silanization on the behavior of a polymer matrix composite. Therefore, the present work characterizes for the first time the effect of silanization pretreatment on the chemical impact and thermal properties of a polyester matrix composite reinforced with 30 wt.% sponge gourd. Both composites with and without (control) silane-treated sponge gourd fibers were characterized by X-ray diffraction (XRD), Fourier transformed infrared spectroscopy (FTIR), thermogravimetry (TGA), and Charpy impact tests, as well as being analyzed via scanning electron microscopy (SEM) coupled with energy-dispersive X-ray spectroscopy (EDS). These characterization techniques aim to reveal the composite’s potential for engineering application.

## 2. Materials and Methods

### 2.1. Materials

The sponge gourd fibers were obtained from a local supplier in the city of Alegre, in the state of Espirito Santo, Brazil. The as-received fibers of variable sizes were cleaned to remove dirt and dried in an oven with air circulation at 100 °C for 24 h. The fibers were hand-laid in a mold so that most of them to be aligned in the loading direction might the composites, as shown in Figure 1b.

The polyester resin (UC 2120 AC PLUS), with a molecular weight of Mn = 9 × 10^3^ g/mol, as well as the butanox catalyst (M-50 (São Paulo, Brazil) were supplied by Redelease (Brazil).

### 2.2. Methods

#### 2.2.1. Chemical Treatment: Silanization

The silanization treatment was based on the methodology used by Bonelli et al. [30]. The sponge gourd fibers after being washed in water and dried in an oven with air circulation at 100 °C for 24 h were treated with 3-methacryloxypropril-trimethoxy-silane provided by SIGMA-ALDRICH (Saint Louis, MO USA). In this process, the fibers were directly impregnated by hand-stirring with silane at a 1:3 (*w*/*w*) silane/fiber ratio and diluted in 1 L of acetone until the acetone evaporated completely to ensure their complete wettability. Subsequently, they were dried in an oven with air circulation at 100 °C for 5 h.

#### 2.2.2. Preparation of the Composites

The manufacturing process for the composites followed the methodology described by Maradini et al. [19], and the samples were produced according to a standard procedure [31]. The composites were manufactured with 30 wt.% sponge gourd incorporated into a polyester matrix. The catalyst was added at 2 wt.% in the resin, as suggested by the manufacturer. Subsequently, the fibers were inserted into a silicone mold with shapes and dimensions according to ASTM D6110 [31]. The resin mixture was poured into this mold and placed in a compressed air reactor MM Company (Lavras, Brazil). After 24 h of curing under 90 bar of pressure, the complete polymerization occurred.

#### 2.2.3. X-ray Diffraction and Morphological Characterization

The X-ray diffraction (XRD) pattern was recorded using a Rigaku MiniFlex 600 Diffractometer (Tokyo, Japan) with Cu Kα radiation (λ = 1.54 Å). The samples were scanned at 2θ range 3° to 70° with a step size and scan rate of 0.05° and 2° min^−1^, respectively. The fiber crystallinity index (CI) was calculated from the empirical method proposed by Segal et al. [32], according to [33]:

(1) $$CI=\frac{I_{\left(200\right)}-I_{\left(am\right)}}{I_{\left(200\right)}}\times 100$$

where *I*_(200)_ is the maximum intensity of the main diffraction peak related to the crystalline plane with diffraction at 2θ = 22° and 20°. and *I*_(*am*)_ represents the amorphous halos at 2θ = 18° and 16° for celluloses I and II, respectively [34]. This method was chosen because it is commonly employed in cellulose-based materials [33]. The crystallinity index for the composites was calculated via the superpositions of crystalline and amorphous areas, using Match 3 software demo version (Bonn, Germany).

#### 2.2.4. Fractographic Analyses

The fracture regions of the composites and the fiber surfaces were observed using scanning electron microscopy (SEM) with secondary electrons in a model JSM-IT200 instrument from JEOL (Tokyo, Japan). The samples had been previously coated with gold. The chemical influence of the silane treatment on the fiber surface was analyzed via energy-dispersive X-ray spectroscopy (EDS).

#### 2.2.5. Chemical Characterization

The contents of lignocellulosic materials present in the fibers, both in natura and after the silanization, were assessed according to the methodology adapted by Gonçalves et al. [35]. The ash content of the material was determined using the TAPPI T211 cm-02 standard. The determination of extractives was performed in two cycles: (i) a solution of toluene and ethanol as a solvent (2:1) for the first 6 h of extraction; (ii) only ethanol for the second extraction for 4 h, according to the TAPPI T204 cm-97 standard. The free extractives material was used for the determination of the Klasson lignin, holocellulose, and cellulose contents according to TAPPI T222, TAPPI T203 cm-09, and TAPPI T203 cm-99 standards, respectively. The percentage of moisture present in each sample used for the characterization tests was calculated from 1 g of material in the moisture analyzer Shimadzu, model MOC63u (Tokyo, Japan), located in the Chemical Engineering Laboratory 3, UFES-Alegre.

#### 2.2.6. Fourier Transform Infrared Analysis (FTIR)

The identification of the fibers’ chemical components, both in natura and treated, as well as the fiber-reinforced composite, was done using Fourier transform infrared (FTIR) spectroscopy. The spectra were obtained with a Bruker spectrophotometer, model Tensor 27 (Billerica, MA, USA), using the attenuated total reflection (ATR) technique with mid-infrared region scans (400–4000 cm^−1^).

#### 2.2.7. Charpy Impact Test

**Figure 2:**
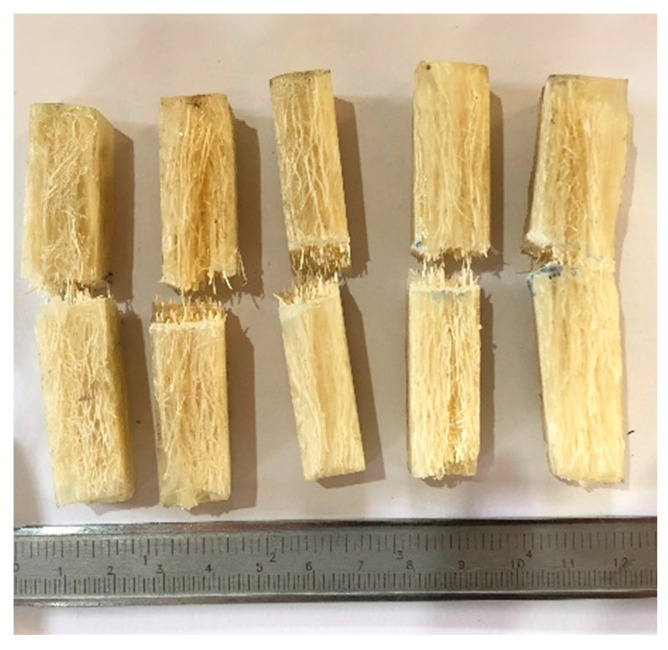
Polyester composites reinforced with 30 wt.% sponge gourd fibers after the Charpy impact test.

Charpy impact tests were performed in a Pantech Instruments system (Gujarat, India) with a pendulum of 11 J. Notched composite specimens with prismatic standard dimensions and a depth of 2.54 mm and angle of 45° were fabricated using a manual notcher (CEAST, model Notchvas), following the ASTM D6110 standard [30]. Five samples were produced for the 30 wt.% fiber concentrations Figure 2.

#### 2.2.8. Thermal Analysis

The thermogravimetric analysis (TGA) and its derivative thermogravimetry curves (DTG) were carried out using a LabSys Evo Thermogravimetric Analyzer (Caluire, France) from room temperature to 700 °C with a heating rate of 10 °C min^−1^ under an inert nitrogen atmosphere to evaluate the thermal degradation of the fibers and composites in all conditions.

## 3. Results and Discussion

### 3.1. Lignocellulosic Characterization

Table 2 presents the results obtained for the chemical characterization of the fibers without treatment and after the proposed treatment. The 66% increase in lignin content for the silanized fibers when compared to the fibers in natura can be associated with the increase in mechanical properties, since lignin plays an important role in the fiber strength, as well as the greater impermeability and stiffness (GONÇALVES et al., 2021). It can also be observed that there is an increase in the alpha cellulose content, which does not degrade, of 8.7% for the silanized fiber. This result is in line with the purpose of the composites, since for the production of polymeric composites it is essential that that lignocellulosic materials have the lightest possible cellulose content.

### 3.2. X-ray Diffraction (XRD)

XRD was used to evaluate how the silanization treatment affects the crystallinity of the fiber cellulose, since the crystalline cellulose is produced naturally [34]. Unlike the mostly amorphous hemicellulose and lignin, cellulose has a semi-crystalline structure, due to the hydrogen bonds and van der Waals forces that exist between its molecules [35].

**Figure 3:**
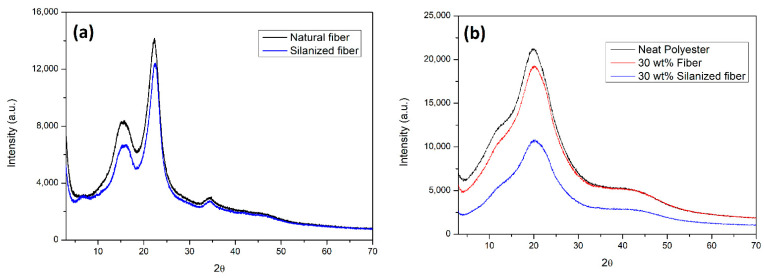
Comparative XRD curves for (**a**) in natura and silanized sponge gourd, (**b**) neat polyester. and composites reinforced with 30 wt.% fiber with and without treatment.

The XRD profiles for both the in natura and silanized sponge gourd fibers are presented in Figure 3a. Figure 3b shows the XRD profiles for the neat polyester and 30 wt.% reinforced polyester composites with and without silanized sponge gourd fibers. From Figure 3a, one can notice only the presence of type I cellulose, as verified by the two well-defined characteristic XRD reflections. One halo of a lower intensity can be seen at 2θ = 15.6°, which refers to the crystallographic plane (110). A second peak of a greater intensity at 2θ = 22.3°, corresponding to the (002) plane, indicates a greater amount of crystalline material in this position [20,35]. A third peak, which is almost imperceptible in Figure 3a, can be identified at 2θ = 34.8° (040) [35].

As expected, the XRD patterns for the composites exposed in Figure 3b exhibit peaks at 2θ = 12.17, 19.79, and 40.92°, corresponding to the crystalline planes at (110), (210), and (111), respectively, which are characteristic of type II cellulose [34].

Table 3 shows the crystallinity index (CI) of each sample that was analyzed. It is possible to observe an increase in crystallinity due to the silanization. There is an increase of 6% in the fiber crystallinity after the chemical treatment, while for the composite with 30 wt.% *Luffa cylindrica* fibers (LCF) the crystallinity increases by 7%. However, when 30 wt.% silanized fibers are added, the CI increases by 47%, results similar to those reported by Teixeira et al. [25]. The silane treatment chemically modifies the fibers, removing the amorphous components and increasing the percentage of crystalline material. In addition, it defibrillates the fibers, which increases their surface area. This is the expected result of the contact with the polymer matrix [25].

The crystallinity of cellulose is an important condition for the definition of its suitability as a reinforcement, as well as its thermal and mechanical resistance in the engineering of composites [36]. The obtained results indicate that the silanization treatment was effective in increasing the crystallinity of the composites and the fibers, which is important for their use as reinforcements, giving more rigidity and being able to reach a higher Young’s modulus [34,36].

### 3.3. Fourier Transform Infrared Analysis (FTIR)

**Figure 4:**
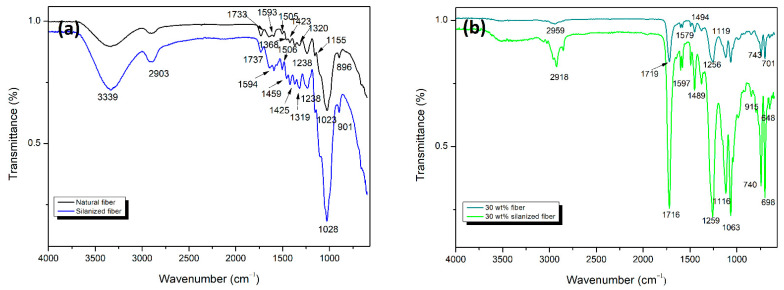
FTIR spectra of (**a**) natural and silanized sponge gourd fibers and (**b**) polyester composites reinforced with 30 wt.% of natural and silanized sponge gourd fibers.

A more detailed study on the composites with both in natura and silanized sponge gourd fibers was obtained using the FTIR technique, as shown in Figure 4. The internal absorption of 3344 cm^−1^ of the in natura fibers (Figure 4a) demonstrates the presence of hydroxyl groups (OH) with strong intramolecular hydrogen bonds [35]. These bonds are related to the lignocellulosic material that makes up the cellulose; in certain cases, because of the crystalline structure, the hydroxyl group is not bonded to the hydrogen bond [19,35].

The band at 2900 cm^−1^ corresponds to the C-H bonds, which are aspects of the organic molecules in natural components. The band at 1733 cm^−1^ is associated with the OH groups and water molecules that are absorbed [19]. It is possible to relate the bands at 1423 and 1320 cm^−1^ to the angular deformation in the plane of the CH_2_ group and the angular symmetric deformation in the plane of the CH_2_ groups, respectively. The band at 1368 cm^−1^ is related to C-H deformation, while the band at 1155 cm^−1^ is associated with the angular deformation of the C-O bonds of the esters in the fibers. The purity of the crystalline cellulose is indicated by the bands at 1023 and 896 cm^−1^, while the 1023 cm^−1^ band is caused by stretching of the C-O bonds and alteration of the alkoxide groups. The band at 896 cm^−1^ is linked to the axial strains of C-O-C bonds and β-glycosidic bonds existing in the glucose groups of the cellulose [19,35].

It can be seen that there were changes in the wavelengths of the FTIR spectra after the silanization treatment, indicating stretching between the Si-O-Si bond and the Si-O-cellulose bonds. The bands at 901 and 1157 cm^−1^ (Figure 4a) confirm the binding of cellulose to silane, as well as the stretching of the bands that are visible at 1023 cm^−1^. The bands at 1238 to 1319 cm^−1^ also confirm the binding of Si-O-Si and Si-O-cellulose [37].

The spectra found in the analysis of composites reinforced with 30 wt.% silanized and natural fibers (Figure 4b) showed bands characteristic of polyester without reinforcement, similar to that found by Maradini et al. [19], on the spectra of the neat polyester and its composites reinforced with cellulose nanocrystals. The bands at 2918, 2953, and 2959 cm^−1^ are associated with C-H stretching and the band at 2369 cm^−1^ refers to the CO_2_ from the atmosphere of the analysis. The bands at 1716 and 1719 cm^−1^ represent the carboxyl group (C=O), and the composite strengthened with 30 wt.% silanized fibers showed a band of greater elongation. The spectra at 1259 and 1116 cm^−1^ are due to the C-O-C stretching vibrations attached to the aliphatic and aromatic groups, respectively. The bands at 1063 and 698 cm^−1^ are possibly associated with the in-plane C-H and C=C aromatic rings, respectively [19].

Considering these results, it can be observed that there was no change in the chemical structure. However, there were small variations in the frequencies of absorption bands of composites reinforced with in natura or silanized sponge gourd fibers, which might be attributed to an enhancement in the silanized fiber adhesion to the polyester matrix. These modifications can attest to the interactions of the reinforcement with the matrix [19].

### 3.4. Charpy Impact Test

**Figure 5:**
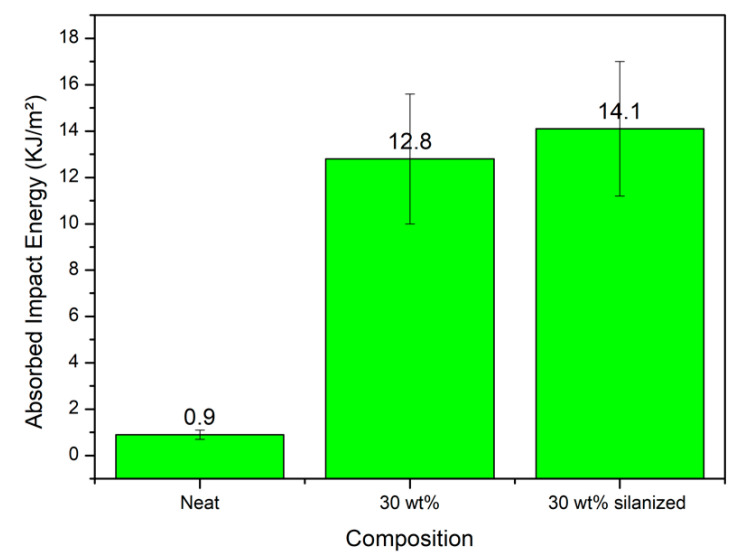
Variation of the absorbed impact energy of the neat polyester and composites reinforced with 30 wt.% sponge gourd fiber contents.

The Charpy impact test was performed to observe the energy variations in the neat polyester matrix as well as in the composites, with the addition of both in natura and silanized 30 wt.% sponge gourd fibers. From Figure 5, it is possible to observe that there were significant increases of more than 14 times and 13 times the absorbed impact energy with the addition of 30 wt.% fibers both with treatment and without treatment, respectively.

The results of the present work are superior when compared to those presented by other researchers such as Nazim et al. [38], who evaluated the impact resistance of hybrid composites of glass and kenaf fibers treated with NaOH. They obtained a maximum Charpy impact strength of 8.31 kJ/m^2^ for the polyester hybrid composite reinforced with 20 wt.% treated kenaf and 10 wt.% glass fibers. These results demonstrate that the sponge gourd fibers added to the composites acted as a load transfer mechanism to increase the resistance to crack propagation, thereby improving the toughness of the polyester matrix. Higher values were found for higher fiber ratios using the Izod impact test, as shown in Table 1.

The ANOVA and Tuckey tests, shown in Table 4 and Table 5, respectively, revealed that F_cal_ (30.56) > F_crit_ (2.866), indicating that there was a difference between the absorbed impact energy values of the neat polyester and both composites reinforced with 30 wt.% sponge gourd, both in natura and silanized, with 95% confidence. However, the 30 wt.% composites with and without silanization were statistically equal. The significant increase in the Charpy impact results with the incorporation of silanized sponge gourd fibers can be attributed to the improved interfacial bonds, and consequently a reduction in fiber extractives. These extractives were of an oily and resinous origin, acting as a barrier in bonding the fibers to the polymer matrix [35].

### 3.5. Thermogravimetric Analysis (TGA)

**Figure 6:**
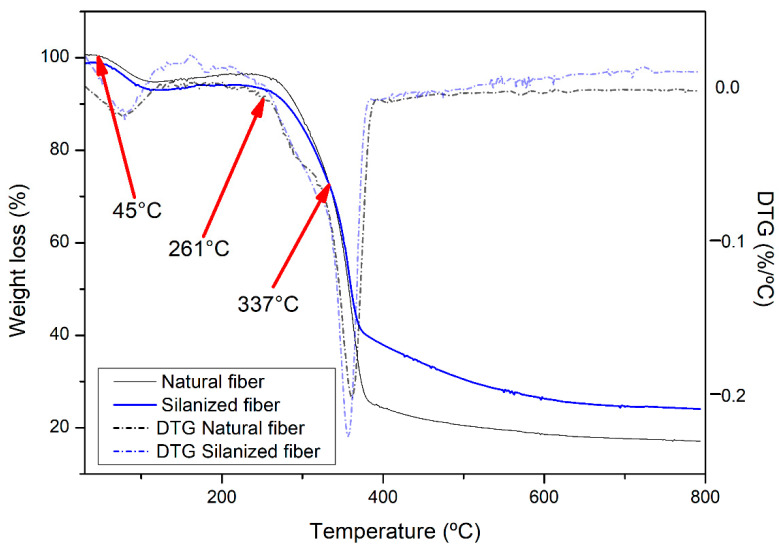
Thermogram (TG) and derivative of thermogravimetry (DTG) for natural sponge gourd fiber and silanized fiber.

**Figure 7:**
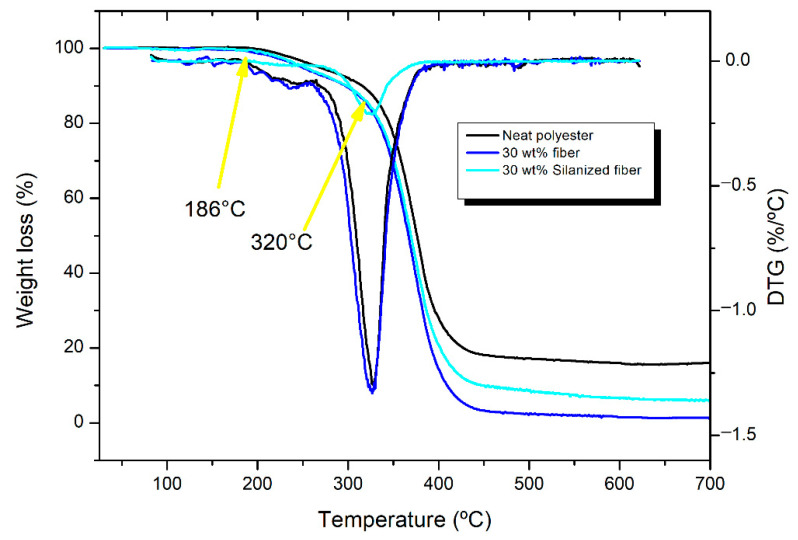
Comparative thermogram and derivative of thermogravimetry (DTG) of neat polyester, whereby the composites were reinforced with 30 wt.% sponge gourd fibers, both without treatment and silanized.

The TGA was performed to investigate the level and thermal stability of the sponge gourd fibers and their reinforced polyester composites with 30 wt.% in natura and 30 wt.% silanized fibers, as shown in Figure 6 and Figure 7. The chemical constituents of the natural fiber cellulose, hemicellulose, and lignin are responsible for the thermal stability of the composite [16]. These constituents have different thermal degradation levels, for which the process is irreversible. Lignin degrades at a temperature range of 160 to 900 °C, and decomposes at a slower rate compared to the other constituents. Hemicellulose decomposes between 220 and 315 °C, and cellulose decomposes between 315 and 400 °C [36].

From Figure 6, it is possible to observe that the degradation of the sponge gourd fiber, both in natura and silanized, occurred in 3 stages. The first stage showed a ~10% loss of mass from 45 °C to approximately 102 °C. This degradation was due to water evaporation [37]. The second stage occurred between 261 and 337 °C, with a mass loss of ~26%, which was related to the breakdown of hemicellulose in the fiber and the cellulose glycosidic bond [36]. The third stage was superimposed on the second stage. From the corresponding DTG, one can observe an acceleration in the weight loss at around 300 °C, which was a result of the third stage. The last stage occurred between 337 and 367 °C, with mass losses of 32% and 46% in the silanized and untreated sponge gourd fibers, respectively, attributed to the breakdown of lignin into subunits [38]. This difference can be assigned to the decomposition of the extractives present in the sponge gourd fiber without treatment.

Based on these results, it is possible to verify that the treated sponge gourd fiber requires a higher temperature than the in natura fiber that is to be degraded, due to the strong intramolecular and intermolecular hydrogen bonds as well as the additional silane molecules, which require more energy for degradation [38]. Normally, the decomposition of cellulosic substances causes a shift to higher temperatures for the treated fibers, thereby promoting an increase in the thermal stability of the composite due to the cellulose chains [36].

The thermal degradation of both the neat polyester and sponge-gourd-reinforced composites is presented in Figure 7, starting near 186 °C and becoming more intense at 320 °C. Up to 200 °C, there is a very low mass loss of <2%, being almost irrelevant. Between 200 and 300 °C, the neat polyester loses 7.67% of its mass, while the composites reinforced with 30 wt.% sponge gourd fibers, with or without silanization, lose 8.55% and 9.12%, respectively. Both composites show good thermal stability up to 300 °C.

The study by Ferreira et al. [39] demonstrated that with the thermal degradation of neat polyester above 100 °C, there is a release of water and a 2% loss at 175 °C, while at 330 °C there is a large loss of mass. Herein, at temperatures above 320 °C, the composites were subjected to an effective thermal degradation process, having a maximum rate at approximately 425 °C. Based on the DTG curves (Figure 7), the neat polyester displays relatively small differences compared to the curves of the composites. The main peaks of the curves show that the composites degraded at temperatures close together, from 327 to 329 °C, while the neat polyester degraded at 327 °C. These results reveal that the incorporation of sponge gourd fibers did not apparently affect the stability of the polyester composites.

### 3.6. Energy-Dispersive X-ray Spectroscopy (EDS)

**Figure 8:**
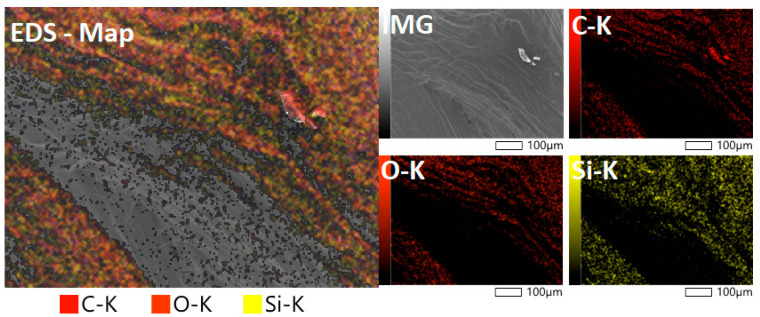
EDS map of *Luffa cylindrica* fibers after the silanization treatment.

Figure 8 presents a micrograph of the fibers after the silanization treatment. The EDS analysis discloses the presence of 49% C, 39.23% O, 8.94% Si, and 2.83% Au, with the gold being due to metallization. The high carbon and oxygen contents are a result of the composition of the lignocellulosic fibers, which consist mainly of cellulose, hemicellulose, and lignin [38]. The silane present in the fiber proves the adhesion of the silane on the cellulose fibers after the chemical treatment.

The oxygen-to-combination ratio (O/C) = 0.80 provides information about the proportion of the composition on the surface; this value indicates that the surface has a lower proportion of lignin, since is O/C ratio is 0.35. The ratio of 0.83 has a relationship with the cellulose, hemicellulose, and pectin. These results point to a higher proportion of these components after the treatment [37].

### 3.7. Scanning Electron Microscopy (SEM)

**Figure 9:**
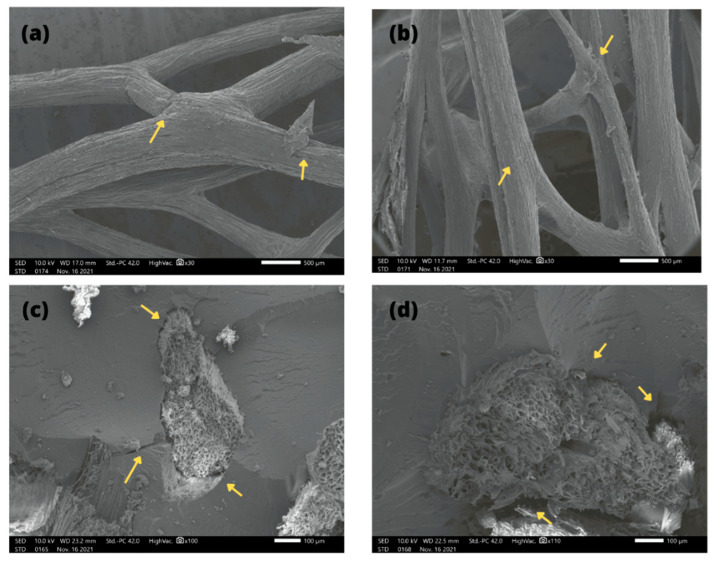
Scanning electron microscopy (SEM) images of (**a**) untreated sponge gourd fibers at 30×, (**b**) silanized fibers at 30×, (**c**) a 30 wt.% sponge gourd fiber-reinforced polyester polymer composite at 100×, and (**d**) a silanized 30 wt.% sponge gourd fiber-reinforced polymer composite at 110×.

Figure 9a–d present the photomicrographs obtained via SEM to analyze the degree of adhesion and compatibility of the polyester matrix reinforced with 30 wt.% sponge gourd fibers, with and without treatment. The photomicrograph of the in natura fiber reveals a typical characteristic of lignocellulosic materials, with rough and irregular surfaces composed of several layers, as indicated by the arrows in Figure 9a. This was also observed in other works [11,15]. After the fiber chemical treatment, one can see a slight increase in roughness (Figure 9b), showing the cellulose microfibrils without any cracking of the material, thereby having a high probability of adhering to the polyester matrix [35].

Figure 9c shows poor adhesion between the fibers and polyester matrix, causing flaws, as indicated by the arrows. These failures are usually observed when the adhesion between the fibers and matrix is not effective, showing that the fiber–matrix compatibility is poor. This result reveals that when a force is applied to the composite, the fibers can be detached from the matrix because the structure of the composite cannot effectively transfer the applied force [40].

The micrograph of the polyester composite with 30 wt.% silanized sponge gourd (Figure 9d) shows a surface with effective adhesion, resulting in a significant increase in polyester–fiber bonding [29]. The silanization of the sponge gourd fibers caused greater activity for the polyester matrix, resulting in an effective formation of covalent bonds between the fiber and the matrix [29]. Owing to this improved adhesion, the fibers are more tenacious under the applied stress and there is no fiber pull-out. Indeed after the impact tests in Figure 5, the silanized-fiber-reinforced composites obtained an improvement of more than 14 times in their impact strength. The interfacial bonds were strengthened, meaning the stress can be transferred without fiber–matrix delamination and fraying [38].

## 4. Conclusions

The addition of 30 wt.% sponge gourd (*Luffa cylindrica*) fibers to polyester matrix composites caused significant changes in the mechanical, thermal, and morphological properties of the composites. The silanization treatment performed on the fibers resulted in composites with superior properties compared to the untreated composites. The SEM results proved the improvement of the fiber–matrix interaction after the chemical treatment, in association with the increased fiber roughness. These results are consistent with the high impact strength value, being more than 14 times that of the neat polyester, as found for the composites with 30 wt.% silanized fiber. Together with the FTIR and TG findings, these results prove the efficiency of the sponge gourd fibers as reinforcement materials in the composites and that the chemical treatment with silane can increase the interfacial adhesion and improve the fiber–polymer thermal behavior.

## Figures and Tables

**Table 1:** Results reported for sponge-gourd-reinforced polymer matrix composites [16].

Polymer	Fiber-Resin Ratio (wt.%)	Impact Strength (KJ/m^2^)	Tensile Strength (MPa)	Flexural Strength (MPa)	Young’s Modulus (GPa)
Polyester	50:50	29.32	46.47	57.36	3.58
Epoxy	13:87	6.67	27.00	55.00	2.41
HDPE	40:60	34.70	20.80	37.70	1.08
Polypropylene	55:45	31.29	37.17	19.40	1.70

**Table 2:** Lignocellulosic compositions of untreated and silanized *Luffa cylindrica* fibers.

	Cellulose (%)	Hemicellulose (%)	Lignin (%)	Extractives (%)	Ashes (%)
Natural fiber	33.36 ± 1.30	38.44 ± 1.30	12.32 ± 0.49	12.15 ± 0.29	0.49 ± 0.05
Silanized fiber	36.26 ± 1.03	32.48 ± 1.03	20.47 ± 2.37	15.01 ± 0.13	8.24 ± 0.15

**Table 3:** The crystallinity index (CI) of natural sponge gourd fibers, silanized fibers, neat polyester, and composites reinforced with 30 wt.% fiber.

LCF/Composites	Crystalline Index, CI (%)
Natural fiber	73.56%
Silanized fiber	77.76%
Neat polyester	6.21%
30 wt.% fiber	6.67%
30 wt.% silanized fiber	9.16%

**Table 4:** ANOVA analysis of the impact strengths of neat polyester and composites reinforced with 30 wt.% in natura and silanized sponge gourd fibers.

	Sum of Squares	df	Mean Square	F	P (Same)	F (Critical)
Between composites	608.1347	4	152.0337	30.587	2.88 × 10^−8^	2.86608
Within groups	99.41067	20	4.970533			
Total	707.5454	24				

**Table 5:** Tuckey test applied via pairwise comparison (Q/p) for the impact strengths of neat polyester and composites reinforced with 30 wt.% in natura of in natura and silanized sponge gourd fibers.

	Neat Polyester	30 wt.% Fiber	30 wt.% Silanized Fiber
Neat polyester		0.000132	0.0001319
30 wt.% in natura fiber	11.95		0.9137
30 wt.% silanized fiber	13.14	1.193	

## Data Availability

The data presented in this study are available on request from the corresponding author.
